# The Antiapoptosis Effect of Glycyrrhizate on HepG2 Cells Induced by Hydrogen Peroxide

**DOI:** 10.1155/2016/6849758

**Published:** 2016-11-06

**Authors:** Miao Su, Tengfei Yu, Hong Zhang, Yan Wu, Xiaoqin Wang, Gang Li

**Affiliations:** Department of Pharmacology, Pharmacy School, Inner Mongolian Medical University, Jinshan Developing Zone, Hohhot, Inner Mongolia 010110, China

## Abstract

This study demonstrated that glycyrrhizate (GAS) could protect HEPG2 cells against damage and apoptosis induced by H_2_O_2_ (1600 *μ*M, 4 h). Cell viability assay revealed that GAS was noncytotoxity at concentration 125 *µ*g/mL, and GAS (5 *μ*g/mL, 25 *μ*g/mL, and 125 *μ*g/mL) protected HepG2 cells against H_2_O_2_-induced cytotoxicity. H_2_O_2_ induced the HepG2 cells apoptosis, obvious morphologic changes were observed after Hochest 33258 staining, and more apoptotic cells were counted in flow cytometry assay compared to that of the natural group. Pretreatment GAS (5 *μ*g/mL, 25 *μ*g/mL, and 125 *μ*g/mL) prior to H_2_O_2_ reverses the morphologic changes and reduced the apoptotic cells in HepG2 cells. GAS reduced the release of MDA, increased the activities of superoxide dismutase, and diminished the release of ALT and AST during oxidative stress in HepG2 cells. After Elisa kit detecting, GAS inhibited the caspase activity induced by H_2_O_2_, GAS decreased the level of caspase-3 and caspase-9 from mitochondria in dose-dependent manner. Western blot results showed that pretreatment GAS upregulated the expression of Bcl-2 and decreased the expression of Bax. These results reveal that GAS has the cytoprotection in HepG2 cells during ROS exposure by inhibiting the caspase activity in the mitochondria and influencing apoptogenic factors of the expression of Bax and Bcl-2.

## 1. Introduction

Oxidative stress is a crucial factor that contributes to aging and multiple degenerative diseases, because it can alter biological molecules such as DNA, proteins, and lipids [1]. Chronic oxidative stress may also promote the onset or progression of chronic liver diseases including nonalcoholic fatty liver disease (NAFLD), cirrhosis, hepatitis, and hepatic carcinoma, which if not treated properly are likely to advance to end-stage liver diseases requiring surgical intervention [2]. Major sources of cellular ROS include the mitochondrial respiratory chain and enzymatic reactions mediated by enzyme systems such as xanthine oxidoreductase, nitric oxide (NO) synthase (NOS), and nicotinamide adenine dinucleotide phosphate (NADPH) oxidases [3]. The physiological and pathological relevance of these sources is different depending on the specific disease and tissue/organ. Superoxide (O_2_^−^) and hydrogen peroxide (H_2_O_2_), primary ROS products, are indeed involved in a broad range disease progress. Previous studies indicated that extracellular H_2_O_2_ increased intracellular ROS levels via multiple mechanisms including loss of intracellular ROS antioxidants such as glutathione (GSH) [4] or decreased mitochondrial membrane permeability followed by mitochondrial ROS release [5]. Many researches have confirmed that the mechanisms underlying various types of hepatic injuries are induced by robust generation of intracellular reactive oxygen species (ROS) [6, 7]. Extracellular H_2_O_2_ has been used to induce oxidative injury in hepatocytes at different concentration [8, 9]. Therefore, H_2_O_2_ was used to induce oxidative injury in HepG2 cells in our study.

Licorice (*Glycyrrhiza glabra* Linn; family: Leguminosae) has been widely used for several centuries in China, especially in Chinese herbal compound. The Licorice has been reported for antipyretic, antimicrobial, hepatoprotective, antioxidant, anxiolytic, expectorant, laxative, and diuretic properties [10–13]. Glycyrrhizate (GAS) was one of the main active ingredients of Licorice. Pharmacology studies in recent years show that GAS has multiple pharmacological effects, including adrenal cortical hormone-like effect, anti-inflammatory antihypertensive effect [14], antiaging and enhancing immune function, the enhancement of body's physiological function, and the inhibitory cancer cell growth. But, few researches on GAS can provide enough data particularly with regard to their mechanism to protect cells against oxidative damage. Here we investigated the effects of GAS on oxidative HepG2 cells damage induced by H_2_O_2_ and elucidated the potential mechanism involved in the cytoprotective effect of GAS.

## 2. Materials and Methods

### 2.1. Chemicals

GAS was obtained from China Institute for Food and Drug control (Beijing, China); the purity of GAS is 98%. Caspase-3 and caspase-9 Elisa kits were purchased from Wuhan Xinqidi Biological Technology Co., Ltd. (Wuhan, china). Hoechst 33258 stains were purchased from Sigma-Aldrich, USA. ALT, AST, MDA, and SOD kits were obtained from the Nan Jing Jian Cheng Bioengineering Institute (Nanjing, China). The tubulin antibody was purchased from Beyotime (Shanghai, china). Annexin V-FITC/propidium iodide (PI) apoptosis detection kit was purchased from BD company (American). Anti-Bax antibody and anti-Bcl-2 antibody were purchased from Abcam company (UK). HepG2 cells were purchased from Beijing Concord Cell Resource Center (Beijing, China).

### 2.2. Cell Culture

HepG2 cells were cultured in a humidified atmosphere of 95% air plus 5% CO_2_ in a 37°C incubator in DMEM supplemented with 10% FBS, 100 *μ*M streptomycin, and 100 U/mL penicillin.

### 2.3. Determination of Cytotoxicity by MTT Assay

Cell toxicity was induced by exposure to H_2_O_2_; cells were challenged with H_2_O_2_ (1600 *μ*M) for 4 hours. The MTT assay is based on the principle that viable cells convert MTT into an formazan crystals, whose absorbance at 490 nm was read in a microplate reader. It has been divided into 5 groups, natural group, H_2_O_2_ group, and GAS (5 *μ*g/mL, 25 *μ*g/mL, and 125 *μ*g/mL). Briefly, HepG2 cells were cultured in 96-well microtiter plates in a final volume of 100 *μ*L culture medium per well. After incubation for 24 hours at 37°C and 5% CO_2_, the GAS groups were pretreated with GAS (5 *μ*g/mL, 25 *μ*g/mL, and 125 *μ*g/mL) for 12 h. After that, the other four groups, except from the nature group, were treated with H_2_O_2_ (1600 *μ*M) for 4 hours. Then, the supernatant was licked off and washed once, 20 *μ*L of MTT (5 mg/mL) in PBS solution was added to each well, and the plate was further incubated for 4 hours. Most of the medium was removed and 100 *μ*L of DMSO was added into the wells to solubilize the crystals. Finally the optical density (OD) was measured by microplate reader at wave length of 490 nm. All viability assays were performed in duplicate; its percentage growth inhibition was calculated using the following formula:

(1) cell viability %=the absorbance of experimental groupthe natural group×100%.

### 2.4. Hoechst 33258 Staining

Briefly, preparations of fixed cells were rinsed three times with PBS, permeabilised with 70% ethanol (30 s), and incubated with a solution of Hoechst 33258 (2 *μ*g/mL) for 30 min at room temperature (RT). Then, the cells were observed by fluorescence microscope (Leica, Germany).

### 2.5. The Detection of ALT, AST, MDA, and SOD

ALT, AST, MDA, and SOD in HepG2 cells were measured with commercial kits according to the manufacturer's recommendations. ALT, AST, and MDA levels and SOD activity were expressed as U/g.prot.

### 2.6. Flow Cytometry

Flow cytometry was performed as described before [15]. Briefly, the HepG2 cells were washed with cold PBS 2 times and then were resuspended in binding buffer (10 mM Hepes/NaOH (pH 7.4), 0.14 M NaCl, and 2.5 mM CaCl_2_), and FITC Annexin V and PI were added. After incubation at room temperature for 15 min in the dark, flow cytometry was analyzed. Annexin V-FITC and PI double staining were regarded as late apoptotic or necrotic cells.

### 2.7. Caspase-3 and Caspase-9 Activity Elisa Kit Assays

Briefly, HepG2 cells were washed twice by PBS, digested with trypsin (0.25%), and collected after centrifugation for 5 mins. Then, the HepG2 cells were lysed on ice (lysate 1 mL : PMSF 100 *μ*L : 11 *μ*L protease inhibitor), the protein was collected, and caspase-3 and caspase-9 were measured with commercial kits according to the manufacturer's recommendations.

### 2.8. Western Blotting Analysis

The expressions of Bcl-2 and Bax were detected by Western blotting. HepG2 cells were exposed to 1600 *μ*M H_2_O_2_ with or without GAS. Cytoplasmic extracts were prepared with 150 *μ*L cell lysis buffer (lysis buffer : phenylmethylsulfonyl fluoride (PMSF) : protease inhibitor cocktail = 100 : 1 : 1) on ice for 30 min, then the centrifuged supernatant was collected, and the protein concentration was quantified using the detergent-compatible (DC) protein assay kit (Bio-Rad, Richmond, CA, USA). Proteins were mixed with 2x sodium dodecyl sulphate (SDS) sample buffer. A total of 40 *μ*g of proteins were separated in a 10% (w/v) polyacrylamide gel and blotted on a nitrocellulose membrane (Bio-Rad, Hercules, CA, USA). The blots were blocked for 2 h and incubated with polyantibodies Bcl-2, Bax, and tubulin at 1 : 2000 dilution for 12 h. Subsequently, the membranes were washed in buffer (PBS with 0.1% (v/v) Tween-20) and then incubated with horseradish peroxidase link-coupled rat anti-rabbit-antibody at 1 : 1000 in blocking buffer. In all experiments, Ponceau staining was carried out to control equal loading and the bands were visualized by electrogenerated chemiluminescence (ECL) Western blotting system (GE Healthcare Biosciences, USA). Protein levels were also analyzed by ImageJ software. The data shown are representative of at least three experiments.

### 2.9. Statistical Analysis

All data are expressed as mean ± standard deviation (SD) and were analyzed using SPSS 13.0 software (SPSS, Chicago, IL). *p* < 0.05 was considered statistically significant.

## 3. Result

### 3.1. GAS Inhibits H_2_O_2_-Induced Cell Death of HepG2 Cells

**Figure 1:**
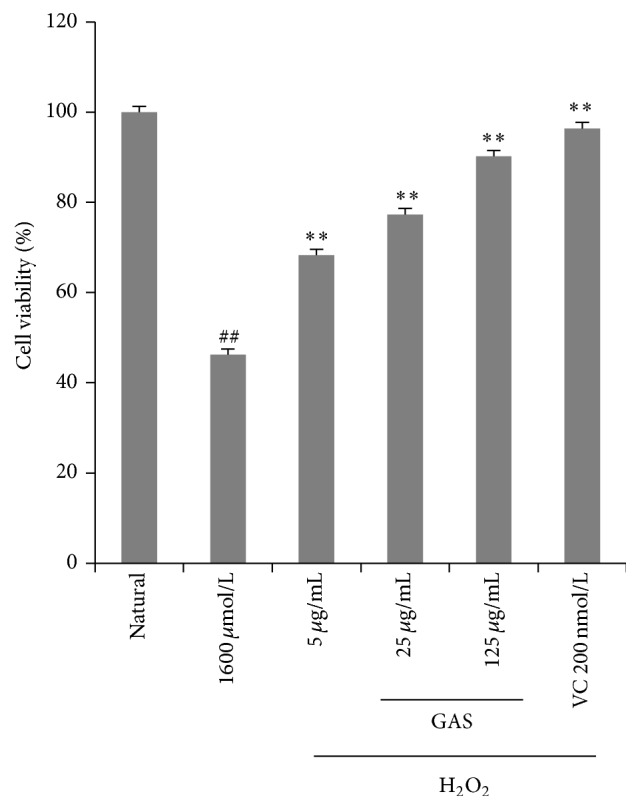
Cell viability of HepG2 cells following different concentrations of GAS pretreatment prior to H_2_O_2_ exposure (1600 *μ*M, 4 h) was measured by MTT assay. With increase of the concentration of H_2_O_2_, cell survival rate was gradually decreased, and with H_2_O_2 _(1600 *μ*M) for 4 hours, its survival rate has been reduced to 52.20 ± 1.13% compared to that of natural group. GAS groups (5 *μ*g/mL, 25 *μ*g/mL, and 125 *μ*g/mL) alleviated the cytotoxicity of HepG2 cells induced by H_2_O_2_, and increased cell viability. The annotation ## indicates a *p* value < 0.05 versus natural group; the annotation *∗∗* indicates a *p* value < 0.01 versus H_2_O_2_ group.

To determine the proper working concentrations of H_2_O_2_, MTT assay was applied to detect the viability that HEPG2 cells were exposed to different concentrations of H_2_O_2 _with 4 h (data not shown). With the concentration of H_2_O_2_ increasing, cell survival rate was gradually decreased, and we found that 1600 *μ*M H_2_O_2_ caused cell viability to decrease by about 50%; its survival rate has been reduced to (52.20 ± 1.13)% compared to natural group. Therefore we exposed HepG2 cells to concentration of 1600 *μ*M H_2_O_2_ for 4 hours to establish an oxidative stress injury model. However, GAS pretreatment groups (5 *μ*g/mL, 25 *μ*g/mL, and 125 *μ*g/mL) effectively protected HepG2 cells from H_2_O_2_-induced cell death (*n* = 6, *p* < 0.01), and GAS alone has no effect on the cell viability of HepG2 cells (Figure 1). Moreover, the antioxidant Vitamin C pretreatment also can reverse the cell death induced by H_2_O_2_, and there is no significant difference between the GAS group (125 *μ*g/mL) and Vitamin C group. The data indicates that GAS, similar to Vitamin C does, effectively protects HepG2 cells from H_2_O_2_-induced cell death.

### 3.2. GAS Inhibits H_2_O_2_-Induced HepG2 Cells Apoptosis

**Figure 2:**
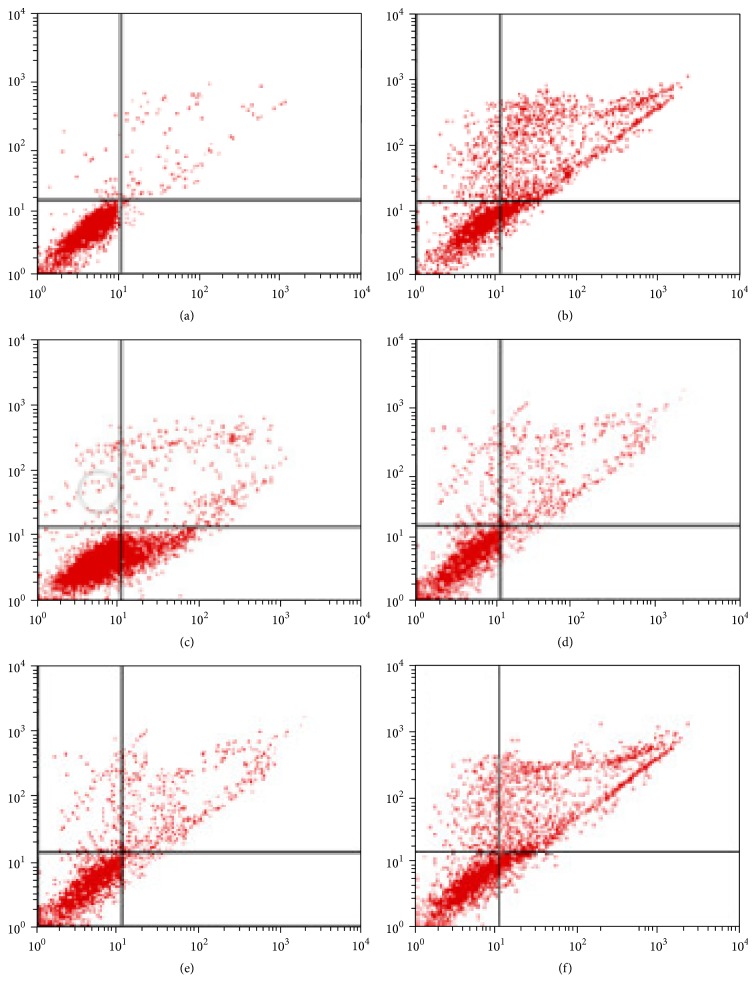
Cells were stained with Annexin V-FITC/PI for verifying the apoptotic or necrotic cell ratio. GAS inhibited H_2_O_2_-induced HEPG2 cells apoptosis. (a) Natural group; (b) H_2_O_2_ group; (c) H_2_O_2_ + Vitamin C (200 nM) group; (d) H_2_O_2_ + GAS (125 *μ*g/mL) group; (e) H_2_O_2_ + GAS (25 *μ*g/mL) group; (f) H_2_O_2_ + GAS (5 *μ*g/mL group).

The Hoechst 33258 staining and Annexin V-FITC/PI double-staining were applied to further evaluate the protective effect of GAS on H_2_O_2_-induced HepG2 cells apoptosis. The results of flow cytometry demonstrated that more apoptotic cells were countered in H_2_O_2_-treated group compared with natural group. The apoptotic percentage in the natural group was 5.30 ± 1.20% (Figure 2(a)), which was significantly reduced than that in the H_2_O_2_ treated group (42.66 ± 1.54%) (*p* < 0.01) (Figure 2(b)). On the contrary, pretreatment of HepG2 cells with GAS (25 *μ*g/mL, 125 *μ*g/mL) prior to H_2_O_2_ showed the significant inhibition on cell apoptosis induced by H_2_O_2_ (*p* < 0.01). The apoptotic percentage in the GAS groups is 19.52 ± 1.19% (GAS 125 *μ*g/mL), 29.31 ± 1.32% (GAS 25 *μ*g/mL), and 34.11 ± 1.44% (GAS 5 *μ*g/mL), respectively, and the apoptotic percentage in the Vitamin C groups is 11.31 ± 2.10%.

**Figure 3:**
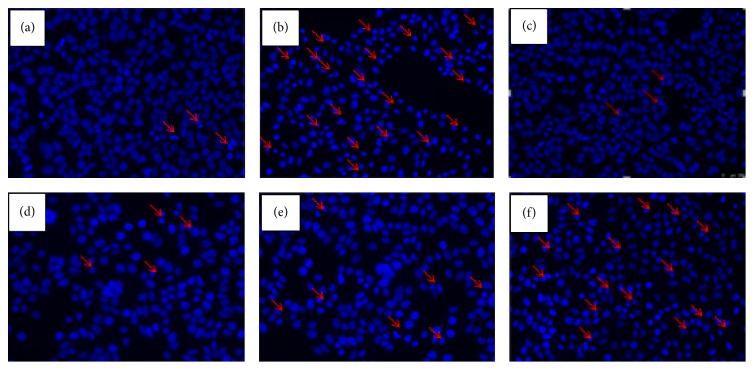
Hoechst 33258 staining indicated that GAS inhibits H_2_O_2_-induced HepG2 cells apoptosis. Morphologic changes in nuclei observed with Hoechst 33258 staining under fluorescence microscopy. (a) Natural group. The HepG2 cells showed normal shape with round intact nuclei; (b) H_2_O_2_ group, HepG2 cells treated with 1600 *μ*M H_2_O_2_ for 4 hours, and the obvious morphologic changes were observed; (c) H_2_O_2_ + Vitamin C (200 nM) group; (d) H_2_O_2_ + GAS (125 *μ*g/mL) group; (e) H_2_O_2_ + GAS (25 *μ*g/mL) group; (f) H_2_O_2_ + GAS (5 *μ*g/mL) group. The arrows indicate apoptotic cells. Original magnification is 400x.

After Hoechst 33258 staining, the HepG2 cells in the natural group showed normal shape with round intact nuclei (Figure 3(a)), whereas the H_2_O_2_-treated cells became more scarce and showed reduced nuclear size, extensive blebbing, strong fluorescent spot, and pyknotic nuclei (Figure 3(b)); these indicate condensed chromatin and apoptotic bodies. The GAS pretreatment groups (25 *μ*g/mL, 125 *μ*g/mL) show protective effect; it altered the morphologic changes in HepG2 induced by H_2_O_2_ (Figures 3(d), 3(e), and 3(f)).

### 3.3. GAS Reduces the Increase of Lipid Peroxidation Induced by H_2_O_2_ in HepG2 Cells

**Figure 4:**
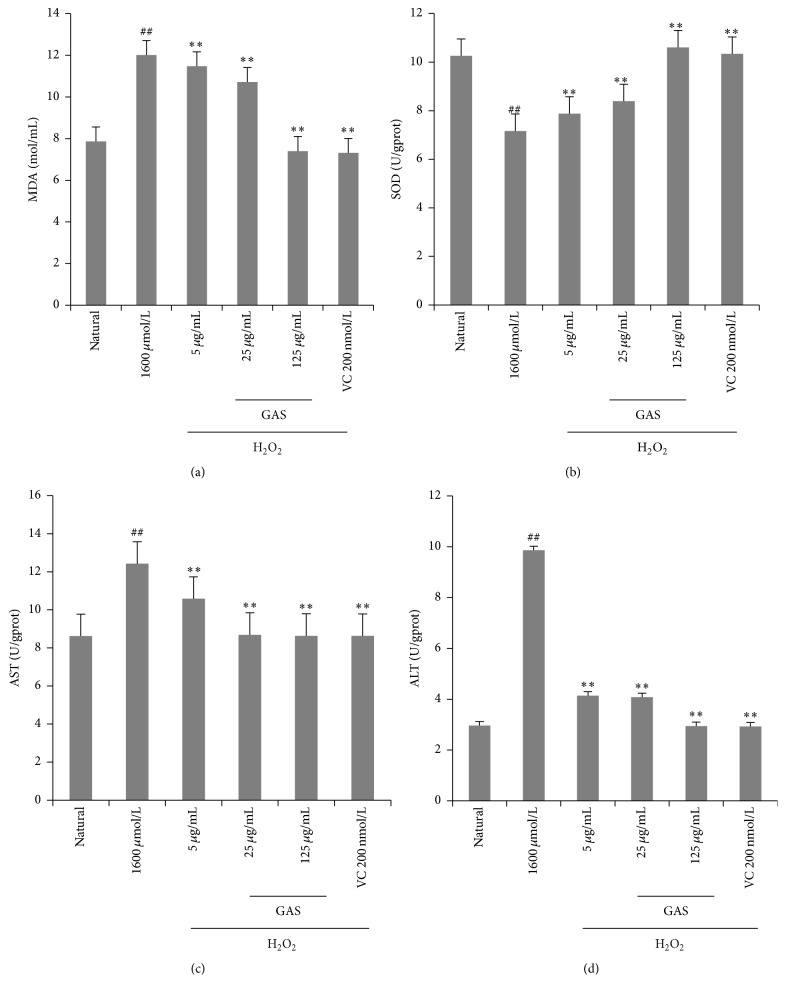
Biochemical assay kits were used to determine content of ALT, AST, MDA, and SOD. GAS protected the cells against H_2_O_2_-induced lipid peroxidation in HepG2 cells and reduced H_2_O_2_-induced reactive protein production. ALT, AST, and MDA content and SOD activity were measured in HepG2 cells. (a) The MDA level in HepG2 cells; (b) the SOD activity in HepG2 cells; (c) the content of AST; (d) the content of ALT. Error bars represent SD (*n* = 6). Experiments were performed at least three times. The annotation ## indicates a *p* value < 0.01 versus natural group. The annotation *∗∗* indicates a *p* value < 0.01 versus H_2_O_2_ group.

Since lipids in cell membrane were prone to oxidation, the effects of GAS in protecting against lipid peroxidation were also investigated. In the study, H_2_O_2_ was used as the source of ROS, which led to oxidative damage in HepG2 cells, and the ALT, AST, MDA, and activity of SOD were measured in the study. The results showed that natural HepG2 cells had little basal intracellular ALT, AST, MDA, and high SOD activity. However, after H_2_O_2_ (1600 *μ*M) exposure to 4 hours, cells had significantly increased intracellular ALT, AST, and MDA accumulation (*p* < 0.01) and significantly reduced intracellular SOD activity (*p* < 0.01). The results confirmed that H_2_O_2_ could induce ROS and led to cell damage in HepG2 cells. GAS pretreatment groups protected HepG2 cells damaged by H_2_O_2_ (1600 *μ*M) (Figure 4) and attenuated the H_2_O_2_-induced changes in ALT, AST, MDA, and SOD activity. HepG2 cells treated with H_2_O_2_ alone evoked a significant increase in the MDA level (Figure 4(a)), approximately 50% higher than the natural group. HepG2 cells treated with GAS showed significant reduction (*p* < 0.05) in MDA levels compared to H_2_O_2_ treated group (Figure 4(a)). In the GAS pretreatment groups (5 *μ*g/mL, 25 *μ*g/mL, and 125 *μ*g/mL), SOD activity was dramatically increased compared with H_2_O_2_ treated group with a dose-effect relationship (*p* < 0.01) (Figure 4(b)). Because of the inhibition of GAS on cell damage induced by H_2_O_2_, we detected less AST and ALT in GAS treated group compared to that of H_2_O_2_ treated group (*p* < 0.01) (Figures 4(c) and 4(d)).

### 3.4. GAS Inhibits Activation of Caspase and Expression of Bcl-2 and Bax Induced by H_2_O_2_ in HepG2 Cells

**Figure 5:**
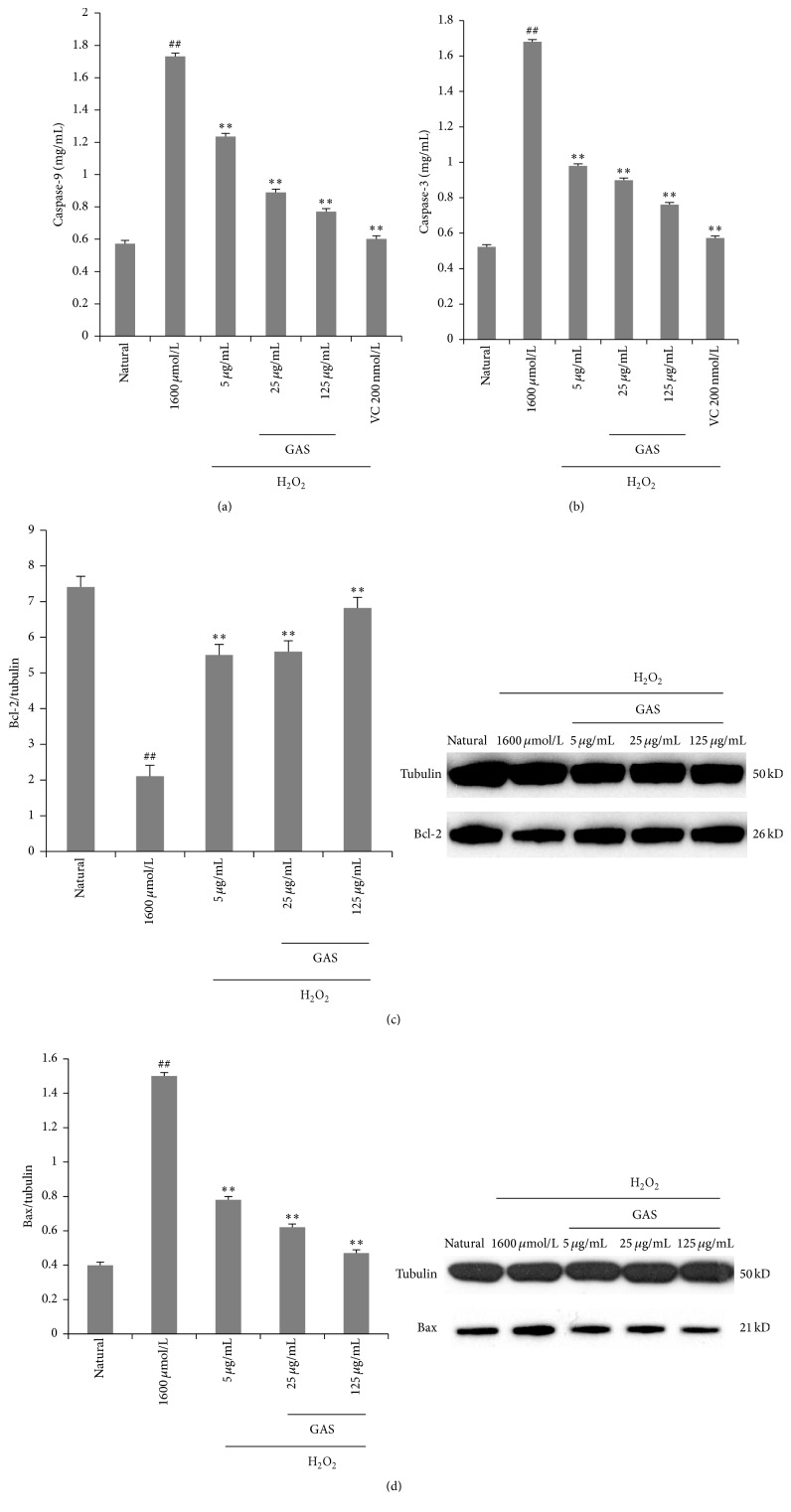
GAS inhibit the apoptosis induced by H_2_O_2_ in HepG2 cells: (a) the activity of caspase-9; (b) the activity of caspase-3; (c) the expression of Bcl-2; (d) the expression of Bax. The annotation ## indicates a *p* value < 0.01 versus natural group. The annotation *∗∗* indicates a *p* value < 0.01 versus H_2_O_2_ group.

As an important modulator of cell death, caspase cascade activation was evaluated in our study. We measured the activities of caspase-9 and caspase-3 by ELISA Kit. As shown in Figures 5(a) and 5(b), caspase-9 and caspase-3 were activated by H_2_O_2_-induced, and GAS effectively inhibited caspase-9 and caspase-3 activation in a dose-dependent manner. These results demonstrated that GAS prevented H_2_O_2_-induced apoptosis through inhibition of mitochondrial-dependent cell death pathways.

The potential role of ROS in Bax and Bcl-2 activation has been indicated in multiple cell death signaling [16, 17]. Bax activation in inducing the cell death by ROS is the critical initiators. Bcl-2 family proteins acts as the gate keepers of cell death at mitochondria, which plays a critical role in regulating Bax induced mitochondrial permeability. As shown in Figures 5(c) and 5(d), Bcl-2 was downexpressed, and the expression of Bax was upregulated after the H_2_O_2_ treated in HepG2 cells. GAS pretreatment group upregulated the expression of Bcl-2 and suppressed the Bax expression with a dose-effect relationship. These results demonstrated that GAS inhibited H_2_O_2_-induced apoptosis through inhibition of Bcl-2 dependent cell death pathways.

## 4. Discussion

In our study, HepG2 cells were used as a cellular model to investigate the effects of GAS on the antioxidant defense systems. We had shown that H_2_O_2_ markedly decreased the viability of HEPG2 cells, whereas pretreatment with GAS significantly inhibited cell injury, as demonstrated by MTT assay. We also detected content of AST, ALT, and MDA, with and without H_2_O_2_; our results indicated that H_2_O_2_ could induce HEPG2 cells damage, which led to the increase of AST, ALT, and MDA and reduction of SOD activity. Oxidative stress caused by ROS is responsible for a wide variety of cellular damage and is the most validated mechanism of secondary injury [18]. Following oxidative stress, the overproduction of ROS and subsequently the depletion of antioxidants resulted in the total breakdown of the endogenous antioxidant defense mechanisms, culminating in failure to protect cells from oxidative damage. Among biomarkers of oxidative stress, MDA and SOD are known as two sensitive indicators [19]. MDA is the end product of lipid peroxidation [20] and MDA levels reflect the extent of cell damage due to oxidative stress. H_2_O_2_ may induce the generation of ROS at mitochondria which has been widely used as a model exogenous oxidative stress mediated experiment in hepatocellular apoptosis [21]. GAS pretreatment effectively protected HEPG2 cells from H_2_O_2_-induced damage. GAS increased the activity of SOD, decreased the level of ALT, AST, and MDA, and inhibited the apoptosis induced by H_2_O_2_.

Apoptosis may be activated by the intrinsic or by the extrinsic pathway [22]. Caspases are a group of aspartate specific cysteine protease, which plays a key role in regulating the apoptosis induced by different kind of stimuli including oxidative stress [23]. Functionally, caspase-3 is an important effector in the apoptotic process, and caspase-9 is an initiator of caspase-3 in the mitochondria-dependent pathway [24]. Treatment of H_2_O_2_ to the HEPG2 cells increased the caspase-9 expression in cells, which indicated that the mitochondrial pathway plays an important function in H_2_O_2_ induced apoptosis in cells. In our study, H_2_O_2_ upregulated the expression of caspase-3 and caspase-9 in HepG2 cells treated with H_2_O_2_; it indicated that the apoptosis induced by H_2_O_2_ through activation of caspases cascade. Bcl-2 proteins are major regulators of mitochondrial cytochrome c and caspases activation. It plays an important role in the regulation of cell apoptosis [25]. This family contains both proapoptotic and antiapoptotic proteins (Bcl-2 and Bcl-XL). Bcl-2 is an important cellular component which can protect against apoptotic cell death. Bax proteins were confirmed that could promote apoptosis. Our study proved that GAS reversed Bax upexpression and Bcl-2 downexpression and suppressed the activity of caspase-9 and caspase-3 in HepG2 cells after exposure to H_2_O_2_. It could be concluded that cell death evoked by H_2_O_2_ is regulated by Bcl-2 family proteins; Bcl-2 downexpression leads to the release of cytochrome c from the damaged mitochondria, which then binds to the adaptor molecule APAF-1 and an inactive “initiator” caspase, procaspase-9, within a multiprotein complex called the apoptosome. This leads to the activation of caspase-9, which then triggers a cascade of caspases activation (caspase-3 and caspase-7) resulting in the morphological and biochemical changes associated with apoptosis. The Bcl-2 upexpression of GAS may be the key mechanism for antiapoptosis induced by ROS in HepG2 cells.

In summary, the present study shows that H_2_O_2_ can induce HepG2 cells injury and induce cells apoptosis. GAS protects human HepG2 cells against H_2_O_2_-induced oxidative stress, cells apoptosis, ROS activity, and activities of caspase-9 and caspase-3. GAS also can regulate the expression of Bcl2 and Bax. GAS-mediated protection can be conferred by one or more of the following mechanisms: GAS could reduce the oxidative stress injury. Second, GAS could attenuate apoptosis through inhibiting the subsequent biochemical changes in the Bcl-2 apoptotic pathway. These data help explain the protective action of GAS against cell injuries involving the mitochondrial pathway.
